# Palisaded Encapsulated Neuroma of the Trunk: A Case Report and Review of Palisaded Encapsulated Neuroma

**DOI:** 10.7759/cureus.726

**Published:** 2016-08-08

**Authors:** Bryce Beutler, Philip R Cohen

**Affiliations:** 1 University of Nevada, Reno School of Medicine; 2 Department of Dermatology, University of California, San Diego

**Keywords:** circumscribed, neuroma, neurofibroma, palisaded encapsulated neuroma, pen, schwannoma, solitary circumscribed neuroma

## Abstract

Palisaded encapsulated neuroma is a rare, benign cutaneous tumor. It most commonly presents as a solitary, flesh-colored, dome-shaped nodule affecting the face. However, albeit rarely, palisaded encapsulated neuroma may also appear on the trunk, genitals, or extremities. We describe the clinical and pathologic findings of a male patient who presented with a palisaded encapsulated neuroma on his left flank. In addition, we review the characteristics of patients with truncal palisaded encapsulated neuromas and summarize the clinical and histologic differential diagnosis of this tumor.

## Introduction

A palisaded encapsulated neuroma is a benign cutaneous neoplasm that most commonly presents as a painless, dome-shaped, flesh-colored nodule or papule. The lesions typically appear on the face; however, albeit rarely, they may also develop on the extremities, trunk, or genital region. Although more patients present with solitary lesions, some individuals present with multiple lesions [1]. The distinguishing histologic feature of a palisaded encapsulated neuroma is an interweaving pattern of benign spindle cells in the dermis.

The clinical and pathologic findings of a 65-year-old male who developed a palisaded encapsulated neuroma of the trunk are described. In addition, the characteristics of other patients with truncal palisaded encapsulated neuromas are reviewed, and the clinical and histologic differential diagnosis for this tumor are summarized. Informed patient consent was obtained for this study.

## Case presentation

A 65-year-old Caucasian male with a history of squamous cell carcinoma presented with a new lesion on his trunk. He had undergone a heart transplant approximately two years earlier. He was being treated with sirolimus (Rapamune) (2 mg per day) and tacrolimus (Prograf) (5 mg per day).

**Figure 1:**
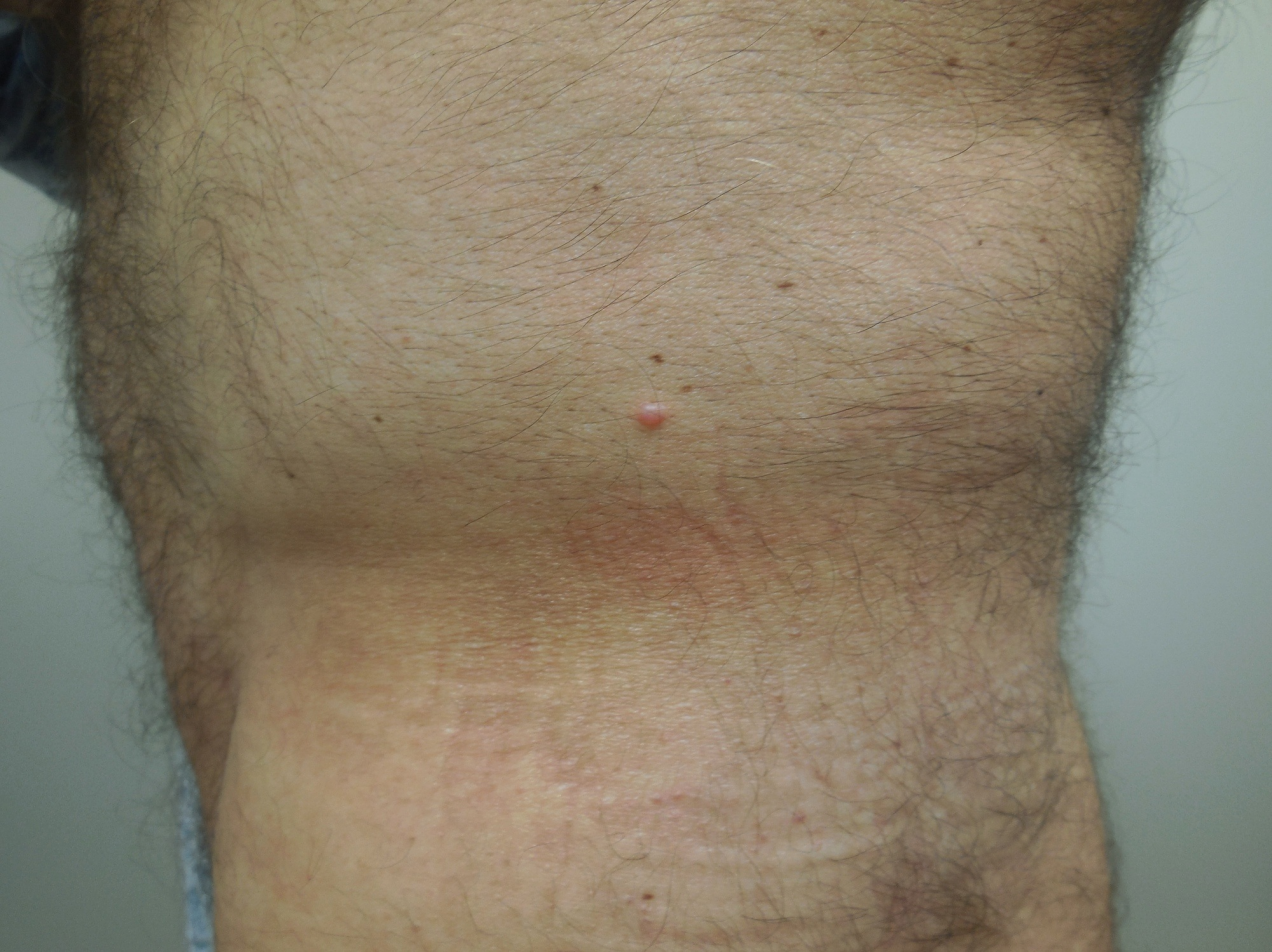
A distant view of a palisaded encapsulated neuroma on the left flank of a 65-year-old Caucasian male

**Figure 2:**
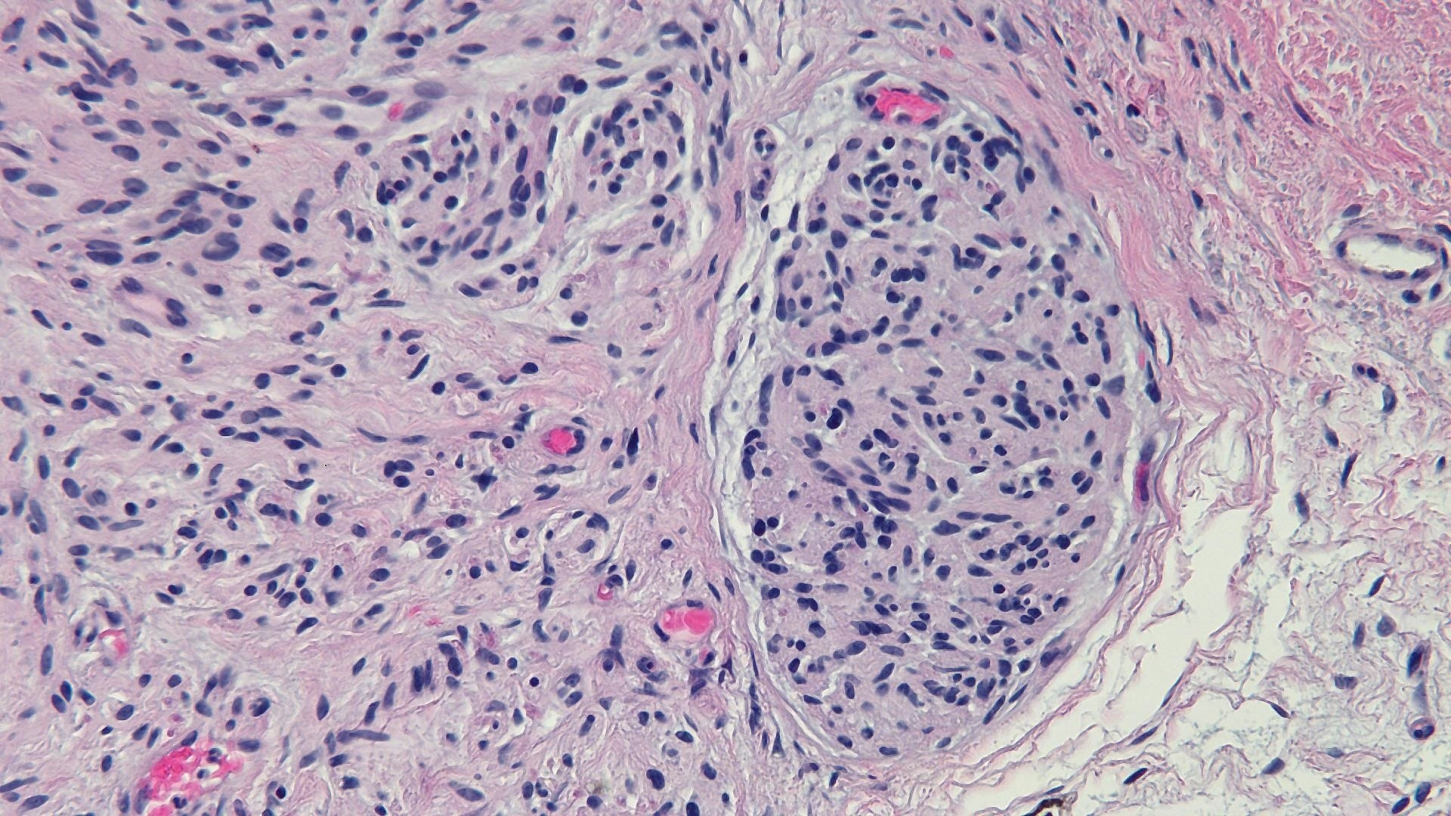
A high magnification view of the microscopic changes noted on a palisaded encapsulated neuroma from a tissue biopsy of a 65-year-old male's left flank tumor Fascicles of benign-appearing spindle cells with palisading of their nuclei are observed in the upper dermis. There are also clefts between the neural cells and the surrounding stroma. (Hematoxylin and eosin; x20)

A cutaneous examination revealed a 3 x 3 millimeter dome-shaped, flesh-colored papule on his left flank (Figure 1). A microscopic examination of the tissue specimen revealed a benign neoplasm of neural differentiation. There were fascicles of benign-appearing spindle cells with palisading of their nuclei. There were also clefts between the cells and the surrounding stroma (Figure 2).

Based on the correlation of the clinical presentation and histopathologic findings, a diagnosis of palisaded encapsulated neuroma was established. The lesion was completely removed by performing a shave biopsy. No further treatment was necessary.

## Discussion

Palisaded encapsulated neuroma was first identified in 1972 by Reed, et al. [2]. They described a “clinically distinctive, solitary, benign, cutaneous tumor … limited, with rare exceptions, to areas bordering mucocutaneous junctions, predominantly on the face." The condition was attributed to a primary hyperplasia of nerve fibers [2].

Palisaded encapsulated neuromas may occur in both men and women. Reed, et al. observed equal frequency in both sexes [2], but recent evidence suggests that the condition exhibits a slight female predilection [3]. The most common age of presentation is 40 to 60 years. However, children and the elderly may also be affected.

A palisaded encapsulated neuroma most commonly occurs as a solitary tumor on the face. However, individuals presenting with multiple rather than solitary lesions have also been described. In addition, tumors may occasionally develop at uncommon sites, including the hands, feet [3], trunk [1], and glans penis [3].

Multiple palisaded encapsulated neuromas have been observed in both children and adults. In 2010, Moore and White described a seven-year-old female who developed multiple flesh-colored papules on her face and feet that were subsequently identified as palisaded encapsulated neuromas [3]. Three years later, Halder, et al. reported on an otherwise healthy 30-year-old female who presented with zosteriform palisaded encapsulated neuroma affecting her face. This patient had developed a dermatomal distribution of numerous asymptomatic papules that grew in size over a four-year period and were ultimately determined to be large palisaded encapsulated neuromas [4]. Notably, both individuals were otherwise healthy and had no history of trauma to the affected region.

The classic presentation of a palisaded encapsulated neuroma is as a painless, dome-shaped, flesh-colored nodule or papule ranging from 2 to 6 mm in diameter. The lesions may appear similar to other tumors (Table 1) [3-4]. Histologically, a palisaded encapsulated neuroma is characterized by a network of interweaving fascicles of benign-appearing spindle cells in the papillary dermis. Nuclear pleomorphism and mitoses are typically absent. The overlying epidermis is normal [3].

**Table 1:** Clinical differential diagnosis of palisaded encapsulated neuroma [3-4]

Clinical Differential Diagnosis of Palisaded Encapsulated Neuroma
Basal cell carcinoma
Epidermal cyst
Melanocytic nevus
Neurilemmoma
Neurofibroma
Schwannoma
Skin adnexal neoplasm

The histologic features of palisaded encapsulated neuroma may mimic those of other cutaneous neural tumors and smooth muscle tumors, including leiomyoma, neurofibroma, schwannoma, and traumatic neuroma; therefore, immunohistochemical studies may sometimes be required in order to distinguish palisaded encapsulated neuroma from similar-appearing lesions (Table 2) [5]. Spindle cells within palisaded encapsulated neuromas are S-100 positive and glial fibrillary acidic protein (GFAP) negative. Factor XIIIa-positive cells are also frequently present. There is epithelial membrane antigen (EMA) staining of the capsule but not of the spindle cells. Although positive collagen IV staining is common among most cutaneous neural neoplasms, neurofibromas may occasionally be collagen IV negative; therefore, collagen IV staining can sometimes be used to differentiate palisaded encapsulated neuroma from neurofibroma.

**Table 2:** Immunohistochemical features of cutaneous neural neoplasms [5] *Abbreviations: + = present; - = absent; +/- = varies; EMA = epithelial membrane antigen; GFAP = glial fibrillary acidic protein; PEN = palisaded encapsulated neuroma

Antigen	PEN	Neurofibroma	Schwannoma	Traumatic Neuroma
CD34	+/-	+/-	+	+/-
CD57	+	+	+/-	+
CD68	+/-	+/-	+	+/-
Collagen IV	+	+/-	+	+
EMA	-	+/-	-	+
Factor XIIIa	+	+	-	+/-
GFAP	-	+	+	+
S-100	+	+	+	+

Although palisaded encapsulated neuroma most commonly affects the face, it can develop within the oral cavity too. The oral cavity may be the most common site of presentation second only to the face. Our patient developed a solitary palisaded encapsulated neuroma on his left flank. To the best of our knowledge, only seven other individuals with palisaded encapsulated neuroma of the trunk have been described in the literature (Table 3) [1, 6-8].

**Table 3:** Characteristics of individuals with palisaded encapsulated neuroma of the trunk [1, 6-8] *Abbreviations: # Lesions = number of lesions; CR = current report; NR = not reported; Ref = reference Lesions affecting the trunk include those that appear on the back and flank.

Case	Age/Sex	# Lesions	Clinicopathologic Features	Notes	Ref
1	50/F	Multiple	Progressive development over a three-year period of multiple, asymptomatic nodules on the arm and trunk	Sibling (Case 2) developed a similar eruption	1
2	50/M	Multiple	Progressive development over a 15-year period of multiple, occasionally painful papules distributed all over the body including the trunk; a histopathologic examination revealed nodules composed of a lattice-like arrangement of broad fascicles of spindle cells separated by clefts	Both mucocutaneous neuromas and palisaded encapsulated neuromas were present in this individual	8
3	56/M	Multiple	Multiple asymptomatic papules ranging from 2 to 8 mm distributed all over the body, including the trunk; a histopathologic examination revealed nodules composed of intercalated fascicles of spindle cells separated by clefts	Sibling (Case 1) developed a similar eruption	1
4	65/M	Single	A single dome-shaped, flesh-colored papule on the left flank; a microscopic examination revealed a benign neoplasm of neural differentiation featuring fascicles of benign-appearing spindle cells with palisading of their nuclei	None	CR
5	66/F	Single	Palisaded encapsulated neuroma affecting the trunk	Multinodular variant of palisaded encapsulated neuroma	6
6	NR	Single	Palisaded encapsulated neuroma affecting the trunk	None	7
7	NR	Single	Palisaded encapsulated neuroma affecting the trunk	None	7
8	NR	Single	Palisaded encapsulated neuroma affecting the trunk	None	7

Eight patients had palisaded encapsulated neuromas located on their back and flank. One individual (Case 2) presented with both mucocutaneous neuromas and palisaded encapsulated neuromas, and another (Case 5) developed a multinodular variant of palisaded encapsulated neuroma. In addition, two cases (Case 1 and Case 3) describe siblings who presented with similar lesions. This suggests that underlying genetic factors may contribute to the development of palisaded encapsulated neuromas.

The pathogenesis for the development of palisaded encapsulated neuroma is unclear. Reed, et al. postulated that tumors represent an attenuated manifestation of multiple endocrine neoplasia type 2B (MEN2B) syndrome, a genetic disorder characterized by the development of neoplasms in the eyes, mouth, and endocrine glands [2]. However, other authors have observed that palisaded encapsulated neuromas exhibit histologic features distinct from those seen in tumors associated with multiple endocrine neoplasia type 2B syndrome [9]. Furthermore, palisaded encapsulated neuroma frequently occurs among individuals with no family history of multiple endocrine neoplasia type 2B syndrome [9].

A traumatic etiology for palisaded encapsulated neuroma has also been proposed [7]. However, most patients—including ours—did not report any antecedent trauma or injury. Topical sirolimus has been used to promote involution of angiofibromas in tuberous sclerosis patients [10]; whether our patient's chronic immunosuppressant treatment following cardiac transplant promoted the development of his palisaded encapsulated neuroma remains to be determined.

Palisaded encapsulated neuroma is benign. However, a biopsy is typically required to distinguish palisaded encapsulated neuroma from more insidious conditions. Gross total removal of the tumor at the time of biopsy typically serves as an adequate treatment.

## Conclusions

Palisaded encapsulated neuroma is a rare, benign cutaneous condition typically characterized by the development of a solitary, painless, dome-shaped, flesh-colored papule. The lesions most commonly appear on the face but may also affect the hands, feet, oral cavity, and trunk. In rare cases multiple tumors may be present. The cardinal histologic feature of a palisaded encapsulated neuroma is a normal-appearing epidermis with a network of interweaving fascicles of benign-appearing spindle cells of neural derivation in the papillary dermis. Immunohistochemical studies demonstrate cells that stain S-100 positive and glial fibrillary acidic protein negative.

Palisaded encapsulated neuroma most frequently affects men and women between the ages of 40 and 60. However, albeit rarely, it may affect children and the elderly. The mechanism of the development of a palisaded encapsulated neuroma is not fully understood. It may represent a variant of multiple endocrine neoplasia type 2B syndrome. Alternatively, the lesions may develop in response to local microtrauma. Palisaded encapsulated neuroma is benign. However, care must be taken to distinguish this tumor from other neoplasms. Treatment often involves resection of the tumor during biopsy.
